# Development of an Imaging‐Based Method for Analyzing Voided Urine Flow Using Conventional and High‐Speed Video Cameras: A Phantom Study

**DOI:** 10.1111/luts.70084

**Published:** 2026-07-28

**Authors:** Masahiro Goto, Tomonori Minagawa, Hiroaki Hara, Noriyuki Ogawa, Tetsuichi Saito, Tetsuya Imamura, Manabu Ueno, Yoshiyuki Akiyama

**Affiliations:** ^1^ Department of Urology Shinshu University School of Medicine Matsumoto Japan

**Keywords:** flow‐dynamics, lower urinary tract function, male urethra

## Abstract

**Aims:**

This study examined whether voided urine form could be an independent parameter to evaluate pathology of the lower urinary tract separately from conventional uroflowmetric measurements. We also evaluated a novel combined methodology of dynamically measuring the velocity and cross‐sectional area (CA) of voided urine.

**Methods:**

A male urethral phantom was connected to an electric pump, and voided water was recorded using a high‐speed video camera (HSVC). The parameters of voided water form included length to the first twist (LFT), length to the scattered water (LSW), and voided water angle (VWA), which were measured in combination with conventional uroflowmetry. The velocity and CA of voided water were measured using an analysis program of fluid movement. Variations of ductal deformity were simulated using silicon tube attachments.

**Results:**

Each parameter increased with pump speed apart from VWA. Flow rate did not change for any attachment except for the stricture type, for which the relative change of LFT surpassed that of LSW. The VWA results were slightly increased by urethral deformity. All results for velocity and CA increased with pump speed and were unaffected by urethral deformity under fixed water flow.

**Conclusion:**

LFT and LSW represent novel voided water form parameters that are separate from conventional uroflowmetric measurements, such as flow rate. Voided water velocity and CA can be non‐invasively determined by the combination of uroflowmetry and a HSVC.

**Abbreviations**
CA: cross‐sectional area
FPS: frames per second
HSVC: high‐speed video camera
LFT: length to the first twist
LSW: length to the scattered water
LUT: lower urinary tract
RPM: rotations per minute
VWA: voided water angle

## 1 Introduction

Male bladder outlet obstruction is not simply caused by urethral compression due to benign prostatic enlargement. In fact, prostate size does not necessarily correlate with the degree of obstruction, and bladder outlet obstruction is considered to result from a variety of physical and anatomical factors [1, 2, 3, 4, 5, 6, 7]. For example, urethral deformity by an enlarged adenoma can directly increase urine passage resistance via the loss of physiological urethral morphology. Deformity of the urethra can induce flow turbulence, hampering efficient passage through the urethra. Although this concept can be explained by fluid‐dynamical assessment of urine movement inside the urethra, fluid‐dynamical approaches are currently unable to analyze urine passage due to technical limitations.

**FIGURE 1:**
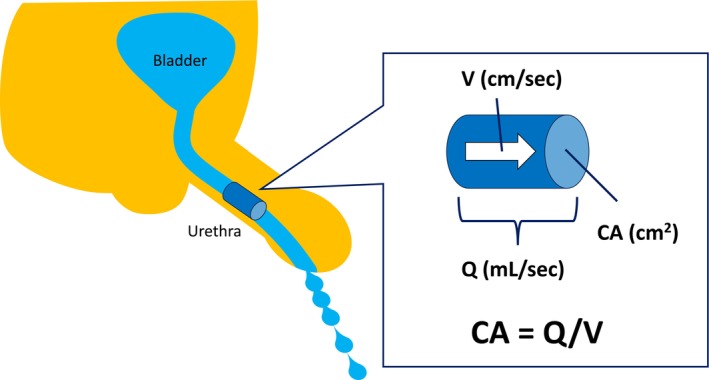
Formula to calculate the CA of voided urine flow (*A*) by dividing quantity (*Q*) by velocity (*V*). *Q* can be measured by uroflowmetry, and *V* can be measured by a high‐speed video camera.

On the other hand, a method for fluid‐dynamical assessment of urine stream and bladder function using a high‐speed video camera (HSVC) combined with ultrasonography has been previously reported [8, 9, 10]. This method demonstrated that the characteristics of the voided urine flow, including vorticity and scattering patterns, vary in accordance with the flow rate. This methodology could be adopted to detect urethral deformity by observing urine flow turbulence. Moreover, an HSVC can measure the velocity of voided urine using a video analyzing program and is able to separately determine the velocity and cross‐sectional area (CA) of voided volume by the formula: CA = *Q*/*V* (Figure 1). The CA of voided urine flow can be calculated by dividing quantity by velocity. Quantity (i.e., voided urine volume; *Q* in the equation) can be measured by uroflowmetry, and voided urine velocity (*V* in the equation) can be identified by our method using an HSVC.

The present study hypothesized that the combination of conventional uroflowmetry and a HSVC could represent a novel technique to evaluate lower urinary tract (LUT) function. However, the mechanisms underlying voiding dysfunction are multifactorial, making it difficult to clarify the pathophysiology or to analyze the influence of individual factors in clinical settings. In the present study, we focused on morphological changes and deformation of the urethra, and employed a simplified and controllable voiding model, although non‐physiological, to facilitate the isolation and analysis of specific pathological factors. We employed a phantom model of the male LUT to assess urethral deformity and water form change suspended in the air, while measuring the velocity of water flow using an analysis program of fluid movement.

## 2 Materials and Methods

### 2.1 Aims and Study Design

The first aim of this study was to confirm whether voided water form in terms of vorticity, scattering, and angle could be parameters for evaluating LUT function and pathology that were separate from conventional uroflowmetric measurements, such as flow rate. Our second aim was to establish a method to dynamically measure the velocity and CA of voided water using a HSVC. Lastly, we evaluated a fluid‐dynamical assessment method of LUT function using a combination of conventional uroflowmetry and a HSVC.

**FIGURE 2:**
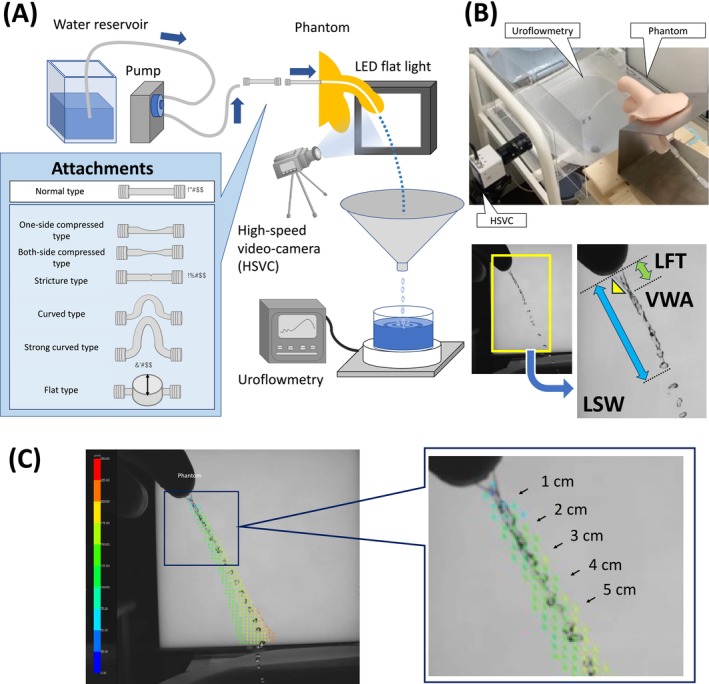
(A) Experimental set‐up of this study. An electric pump was connected to the phantom of a male urethra via attachments devised for simulating urethral deformity. Voided water stream was captured by a high‐speed video camera in front of a LED flat light. The quantity of voided water was simultaneously measured by uroflowmetry. (B) Actual view of the experimental set‐up. Voided water was clearly captured by a high‐speed video camera, and the length to the first twist (LFT), length to the scattered water (LSW), and voided water angle (VWA) were measured. (C) Vector analysis of voided water using flow measurement software. Warm colors indicate increased flow velocity. The velocity of voided water was measured at 1, 2, 3, 4, and 5 cm from the urethral orifice of the phantom.

This phantom study was conducted to explore a new method to detect urethral deformity indirectly using the principles of fluid‐dynamical assessment for LUT function. The phantom was originally constructed using a simulator for the indwelling of a urethral catheter (M200‐7 Male urethral catheterization simulator Plus, Sakamoto Model Co. Ltd., Kyoto, Japan) whose inner urethra diameter was 6 mm. Urethral deformities were reproduced by original attachments made by a 3‐dimensional printer. Voided water from the phantom was simultaneously assessed by a HSVC and uroflowmetry, with water form parameters and velocity measured for each condition (Figure 2A,B). The CA of the voided water flow was calculated by the formula in Figure 1. The changes in water form parameters, velocity, and CA were compared based on the water flow rate for each condition.

### 2.2 Experimental Set‐Up (Pump, Phantom, and Attachments)

A schematic diagram of the set‐up in this study is presented in Figure 2A, with the actual set‐up depicted in Figure 2B. To strictly control flow volume, the male urethral phantom was connected to a tubing pump (Front Lab Tubing Pump FP600‐1515, As one Co. Ltd., Osaka, Japan) through several attachment types. The pump draws water from the reservoir, and pump speed is increased by 50 rotations per minute (RPM) from 200 to 600 RPM in a stepwise fashion. Variations and degree of ductal (urethral) deformity were simulated using hard silicon tube attachments made by a 3‐dimensional printer. The seven types of attachments were as follows: normal, one‐side compressed type (1/3 of one side was compressed), both‐side compressed type, stricture type, curved type, strong curved type, and flat type. Each attachment simulated a clinical urethral deformity as described in “Types of attachments,” and the design of stricture type, curved type, strong curved type, and flat type was shown in Supporting Information File. The one‐side compressed type and both‐side compressed type are modifications of the normal type.

Types of attachments

- Normal: A non‐deformed tube with an inner diameter of 5 mm (14‐16Fr), used as the baseline model and connected to the pump with tubing of the same inner diameter.
- One‐side compressed type: A model in which the lumen was compressed by approximately one‐third from one side, simulating asymmetric urethral compression caused by prostatic adenoma. This model was created by modifying the normal type.
- Both‐side compressed type: A model in which the lumen was compressed by approximately one‐third from both sides, simulating symmetric urethral compression caused by prostatic adenoma. This model was created by modifying the normal type.
- Stricture type: A model simulating urethral stricture with a membranous narrowing, in which the minimum inner diameter was 1 mm.
- Curved type: A model simulating dorsal bending of the urethra caused by compression from an enlarged prostatic adenoma (semicircle of 17.5‐mm radius semicircle).
- Strong curved type: An enhanced version of the curved type with a greater degree of bending, designed to evaluate the effect of increased urethral curvature (the semicircle of the curved type extended outward by 17.5 mm).
- Flat type: A model simulating urethral flattening caused by compression from an enlarged prostatic adenoma (a duct with cylindrical space of 40 mm in diameter and 3 mm in height).

### 2.3 Set‐Up for Observation (HSVC and Uroflowmetry)

The voided water from the male urethral phantom was captured using a HSVC (INFINICAM UC‐1, Photron Co. Ltd., Tokyo, Japan) (Figure 2A). Images were captured at 1000 frames per second (fps) in front of a LED flat light (UFLS‐751, U‐TECHNOLOGY Co. Ltd., Tokyo, Japan) for optimal contrast of the voided water form. In addition, a uroflowmetry device (FLOWMASTER, Labrie Co. Ltd., New Hampshire, USA) was placed under a capturing funnel to collect the voided water.

### 2.4 Measurement of Parameters, Calculation, and Analysis

Referring to the previous report, flow rate (mL/s), the maximal length to the first twist (LFT, pixel), maximal length to the scattered water (LSW, pixel), and maximal voided water angle (VWA, degree) were recorded as parameters of voided water form [10]. The maximum values were used in the present analysis because the parameters examined were strongly dependent on pump rotation speed (RPM), and the peak values most appropriately reflected this dependency. In addition, the water flow exhibited substantial fluctuations, making intermediate or continuous numerical analyses both technically challenging and of limited interpretative value for the purpose of this study. The reference point was defined as the distal tip of the urethral meatus, specifically the proximal edge of the emerging water stream. The reference axis was defined as the vertical line. The direction of the stream was then determined by the straight line extending from the reference point to the LFT, and the angle between this line and the reference axis was defined as the VWA. Additionally, “minimal” values of LFT, LSW, and VWA are measured in this study. Flow rate was simultaneously recorded using uroflowmetry. LFT and LSW were defined as the longest length for 10 s (10,000 images obtained by HSVC) for each condition. Observation of the water stream was performed for 10 s (1 rotation corresponding to 1000 images). This duration was considered sufficient based on observations obtained in a pilot study. Because the measurement conditions remained constant during the observation period, each condition was evaluated using a single measurement. As this experimental setup did not involve measurement variability or inter‐individual differences, statistical analysis was not considered necessary. The observation period of 10 s was determined based on the results of pilot experiments, the recording capability of the high‐speed camera (1000 fps), and the range of pump speeds used in this study (200–600 RPM). Because the experimental model maintained strictly controlled measurement conditions during the observation period, the maximum value obtained from a single measurement was defined as the parameter for analysis rather than performing repeated measurements. LFT and LSW on captured digital images were measured by the PC‐based image analysis software because absolute length of LFT and LSW cannot be directly measured on captured digital images using analysis software (Photron FASTCAM Analysis, Photron Co. Ltd., Tokyo, Japan). To avoid exaggeration, the “pixel” values were not converted to centimeters (cm). VWA was determined using lines between the urethral meatus and maximal LFT. Representative images of LFT, LSW, and VWA are shown in Figure 2B. Following the measurement of each parameter, the percentage of baseline (200 RPM) was analyzed to focus on the relative changes from baseline for each condition. Representative findings of HSVC are presented in Supporting Information Movie.

Before establishing the methodology of measuring the velocity of voided water (cm/s), experimental reproducibility and the exclusion of air resistance against falling water was examined. To determine the optimal position for velocity measurement, the velocity of voided water was determined by flow measurement software (Flow‐PIV, Library Co. Ltd., Tokyo, Japan) at 1, 2, 3, 4, and 5 cm away from the external meatus of the phantom, as shown in Figure 2C. The velocity was calculated as average value from the images obtained for 10 s. Moreover, the flow velocity was calculated relatively based on the phantom's penile diameter being 3 cm. The software can perform particle image velocimetry; a technique used in fluid mechanics to measure velocity fields by tracking the motion of particles in a flow. The measured velocity is demonstrated on a colored scale (Figure 2C). CA (cm^2^) was calculated from the obtained water flow rate and velocity of each length, such as 1, 2, 3, 4 and 5 cm from the external meatus, using the formula in Figure 1. Parameter measurements were done for all attachment types. The results for velocity and CA were compared to evaluate relationship of each parameter.

## 3 Results

### 3.1 Voided Water Form Assessment

**FIGURE 3:**
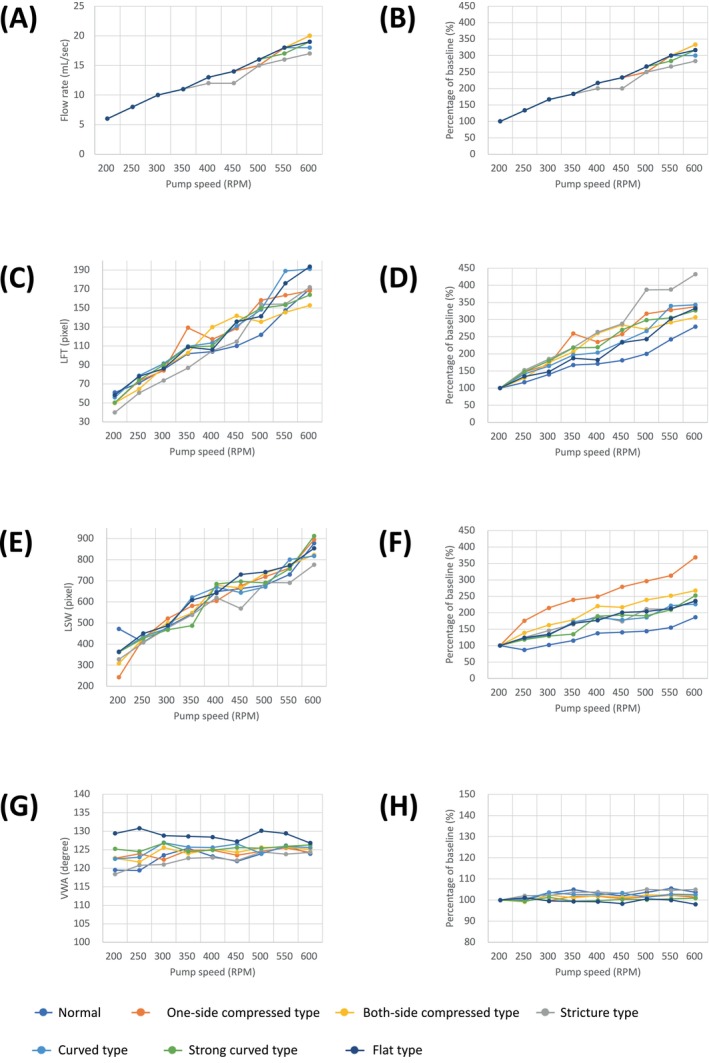
Summarized results of parameters measured by uroflowmetry and a high‐speed video camera at each pump speed setting. The values for flow rate, and “maximal” length to the first twist (LFT), length to the scattered water (LSW), and voided water angle (VWA) are presented in (A), (C), (E), and (G), respectively. The results were graphed for each attachment. The percentage of baseline values at 200 RPM is presented in (B), (D), (F), and (H), respectively.

**FIGURE 4:**
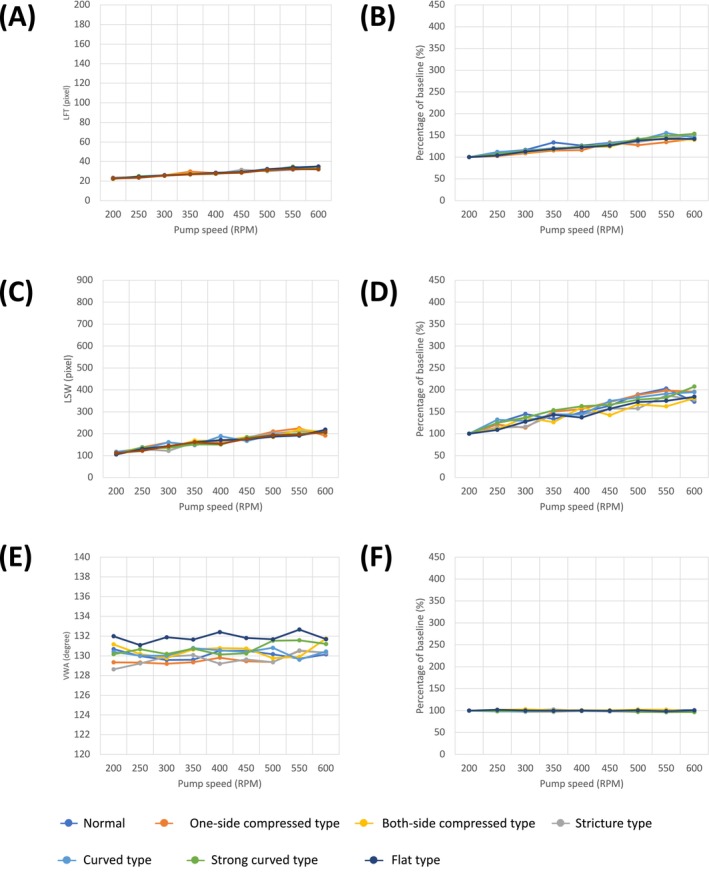
Summarized results of parameters measured by a high‐speed video camera at each pump speed setting. The “minimal” values for length to the first twist (LFT), length to the scattered water (LSW), and voided water angle (VWA) are presented in (A), (C), and (E), respectively. The results were graphed for each attachment. The percentage of baseline values at 200 RPM is presented in (B), (D), and (F), respectively.

The summarized results of the measured values for flow rate, LFT, LSW, and VWA are presented in Figure 3A,C,E,G, respectively. The percentages of baseline value at 200 RPM, indicating the relative change of each parameter, are respectively shown in Figure 3B,D,F,H. All parameters increased with pump speed apart from VWA. Neither the actual value nor the percentage of baseline flow rate changed for any attachment with the exception of the stricture type. Moreover, LFT and LSW changed remarkably for the stricture type attachment, especially for the percentage of baseline value. These results indicated that the morphological findings of voided water were impacted by urethral deformity. In contrast, the minimal value was separately evaluated, and the data could not demonstrate differences among the attachments as shown in the percentage of baseline value shown in Figure 4. Therefore, the maximal value of each parameter should be focused on to analyze voided water flow.

Focusing on attachment type, LFT and LSW were increased in the deformed attachments versus the normal type attachment. The percentage of baseline for LFT increased greater at higher speeds than at lower speeds, with the stricture type showing the strongest LFT change. On the contrary, the percentage of baseline for LSW at 200 RPM changed even at low speeds, and the one‐side compressed attachment demonstrated the strongest change in LSW. The VWA results were also increased by the attachments, with the results of the flat type 10 degrees more than normal conditions. However, the percentage of baseline for VWA did not change remarkably at any pump speed.

### 3.2 Velocity and CA Assessment

**FIGURE 5:**
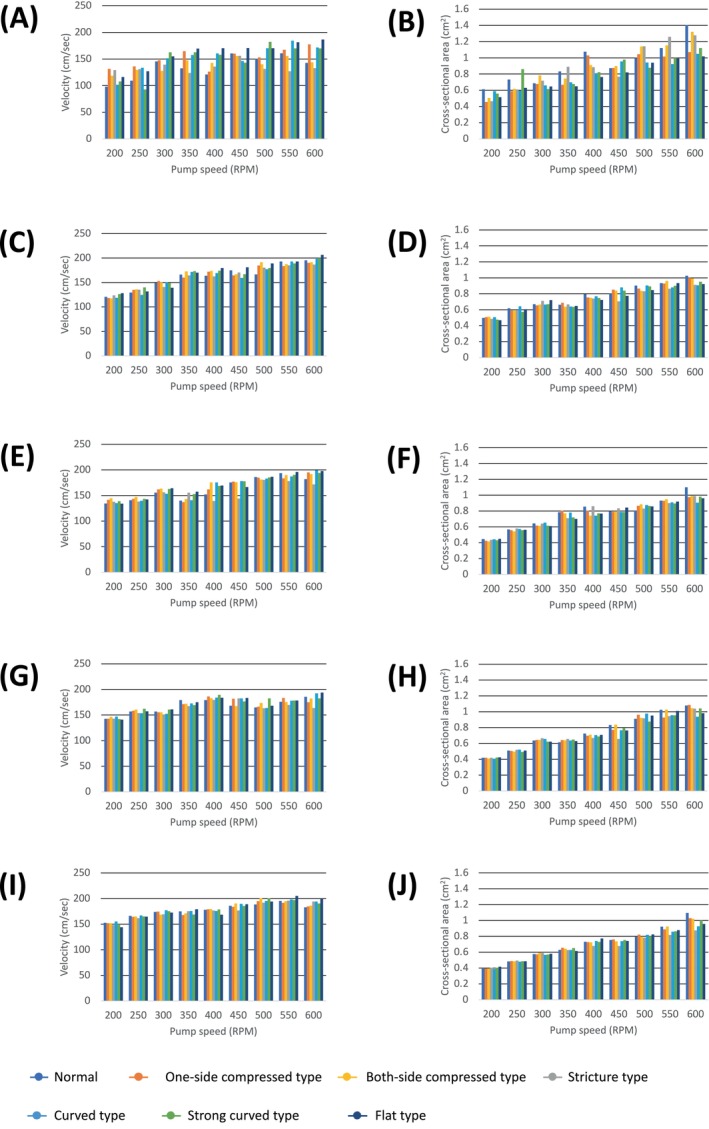
Results for velocity and CA of voided water in this study. Each pump speed and type of attachment were similarly presented as in Figure 3. Velocity was measured at 1 cm (A), 2 cm (C), 3 cm (E), 4 cm (G), and 5 cm (I). CA was similarly calculated at 1 cm (B), 2 cm (D), 3 cm (F), 4 cm (H), and 5 cm (J).

The results for velocity of the voided water are summarized in Figure 5 for 1 cm (A), 2 cm (C), 3 cm (E), 4 cm (G), and 5 cm (I), with those for CA presented for 1 cm (B), 2 cm (D), 3 cm (F), 4 cm (H), and 5 cm (J). All velocity and CA measurements increased with pump speed. Moreover, the type of attachment generally resembled the flow rate results, although both velocity and CA at 1 cm distance (Figure 5A,B) were scattered compared with the other findings. Thus, the obtained and calculated results apart from at 1 cm were similar and confirmed the reproducibility of the methodology for all attachments.

## 4 Discussion

In this phantom study, flow rate was strictly controlled by a positive‐displacement pump in all the attachments which simulate pathologically deformed LUTs. Due to factors such as turbulence within the tubing and air resistance, it is expected that variability in each parameter will be observed even under a fixed flow rate. When the minimum and maximum values were compared, the minimum values were found to have little clinical significance. Therefore, greater emphasis should be placed on the analysis and discussion of the maximum values of each parameter. We observed morphological change of voided water measuring LFT and LSW, and exhibited notable variations among the attachments in spite of the same flow rate as each condition. Especially, LFT at high flow rate and LSW at low flow rate may reflect the effect of urethral deformity outcomes. However, VWA did not show any effects of pump speed changes despite VWA baseline values varying among the attachments. These results indicated that the findings and changes in water form obtained by a HSVC can be influenced by urethral deformity inducing LUT symptoms. According to the continuity equation, voided water amount calculated by velocity and CA theoretically does not change regardless of the presence of stricture in a closed duct. Following this theory, the velocity and CA of the voided water could be measured and did not substantially change at distances greater than 1 cm from the external meatus of the phantom. In addition, the reproducibility of calculating CA was confirmed and found to be independent of urethral deformity.

In the present study, LFT and LSW appeared to exhibit different characteristics. LFT tended to show larger changes at higher flow rates, whereas LSW appeared to change more prominently at lower flow rates. These differences may be influenced by complex fluid‐dynamical phenomena, including jet breakup caused by surface tension (Rayleigh–Plateau instability) and factors related to the Weber number. However, sufficient data to verify these mechanisms were not obtained in the present study, and further investigation will be required.

Evidence on the fluid‐dynamical assessment of the LUT is scarce. Ozawa et al. reported on the velocity of water passing through the male urethra in clinical settings using Doppler ultrasonography and mentioned the significance of the *V* × *A* = *Q* formula [11, 12, 13]. However, urethral compression from outside of the perineum cannot be avoided when using ultrasonography, which can impede urine flow. On the other hand, urethral pressure reflectometry has been employed to measure urethral pressure and CA of the male and female urethra [14, 15, 16]. As urethral catheterization with a pressure introducer must be performed for this procedure, our method represents a useful means to evaluate the velocity and CA of voided urine suspended in the air, without compression of the urethra or more invasive catheterization.

The utility of fluid‐dynamical assessment for LUT function has been assessed mainly in a phantom setting to demonstrate the increased flow resistance caused by urethral deformity using vector flow mapping [17, 18, 19, 20]. These reports indicated the usefulness of a fluid‐dynamical approach inside the urethra for evaluating LUT function and showed that velocity and direction vectors could be valid parameters to measure the characteristics of water passing inside the urethra.

To the best of our knowledge, this study is the first to explore the fluid‐dynamical assessment of LUT function using a HSVC. A simultaneous approach by fluid‐dynamical and conventional methods was successfully established to measure velocity and calculate the CA of voided water. Theoretically, the resolution of velocity (cm/s) can be more precise than that of flow rate (mL/s) determined by uroflowmetry. Considering the potential for fluid‐dynamical assessment of the LUT, our combination method of conventional uroflowmetry and a HSVC may be able to distinguish among urethral obstructions, including benign prostatic hyperplasia and detrusor weakness, without urodynamic study by catheterization.

In normal LUT function, voiding is governed by complex bladder dynamics in which urinary flow gradually decreases from its peak to zero over several to several tens of seconds. In addition, LUT dysfunction involves complex and multifactorial pathophysiological mechanisms, including urethral narrowing, flattening, bending, and impaired urethral relaxation. The present model cannot be considered a comprehensive LUT model that fully reflects such complex voiding dynamics or multifactorial pathophysiology. Nevertheless, isolating specific structural factors—such as a reduction in urethral cross‐sectional area or urethral bending—and examining their effects was considered necessary for the validation of a developing technology such as that investigated in the present study.

Several limitations should be considered when interpreting our results. First, the present experimental setup was intentionally simplified and does not fully reproduce physiological voiding. A rotary pump was used to generate flow because it allows relatively stable and controllable flow conditions with less pulsatility and directional fluctuation than several other pump systems. Nevertheless, this mechanism differs substantially from actual human voiding driven by bladder contraction. In addition, the system employed steady flow under fixed experimental conditions, and the attachments used to simulate urethral morphology were rigid and lacked the distensibility of the urothelium and surrounding tissues. Furthermore, the present model mainly focused on the morphology of the prostatic urethra, although in reality the bladder neck, membranous urethra, and distal urethra may also influence urinary stream characteristics. However, because the present study aimed to establish a fundamental analytical method in a largely unexplored field, such simplification was considered appropriate for evaluating basic flow characteristics under highly controlled conditions while minimizing complex confounding factors. Future studies using more physiologically realistic models, including dynamic urethral properties and broader LUT morphology, will be necessary for clinical translation.

Second, technical limitations of the HSVC system should also be considered. Continuous frame‐by‐frame analysis of all images remains technically difficult because high‐speed imaging at 1000 fps generates a very large amount of data associated with substantial storage and processing burdens. Therefore, in the present study, measurements were obtained from a single standardized 10‐s recording period, and representative maximum values were used for evaluation rather than continuous measurements or averaged values derived from all captured frames. Because the evaluated parameters increased approximately in proportion to pump speed under fixed observational conditions, we considered this analytical approach reasonably valid for comparative assessment. In addition, some parameters related to stream length and morphology were evaluated using pixel‐based measurements rather than actual physical dimensions because direct real‐world measurement of a rapidly moving liquid stream is technically challenging. Although this approach was considered acceptable in a strictly standardized phantom model, future development of automated image‐analysis systems and quantitative measurement techniques capable of obtaining real physical values will be important for clinical implementation.

Third, several methodological limitations related to flow analysis should be considered. The present study evaluated voiding phenomena mainly from a fluid‐dynamical perspective, although the dispersed urinary stream observed after exiting the urethra is also influenced by gravity, viscosity, and other physical factors. In addition, although the theoretical relationship CA = *Q*/*V* is based on the continuity equation, *Q* and *V* were measured at different locations in the present system, which may introduce spatial and temporal discrepancies. Furthermore, the relatively small differences observed among attachment types may have been influenced by several modifying factors, including the unavoidable presence of the penile urethra, the non‐distensible nature of the attachments, and the steady‐flow characteristics of the pump system. These factors may have attenuated the apparent effects of prostatic urethral morphology on urinary stream characteristics. Although the present approach was considered appropriate for a proof‐of‐concept phantom study, future investigations using more advanced synchronous measurement techniques and physiologically dynamic models will be necessary to further validate the clinical significance of this method.

In conclusion, the fluid‐dynamical assessment of voided water using a HSVC represents a novel method to evaluate urethral deformity. The combination of a HSVC and conventional uroflowmetry shows promise as a potential approach for precise, non‐invasive estimation of lower urinary tract function.

## Author Contributions

**Masahiro Goto:** data acquisition and writing‐original draft preparation. **Tomonori Minagawa:** conceptualization, writing‐review and editing, and editing visualization. **Tetsuichi Saito Hiroaki Hara**, **Noriyuki Ogawa**, and **Tetsuya Imamura:** data acquisition. **Manabu Ueno:** data acquisition. **Yoshiyuki Akiyama:** supervision and editing.

## Funding

This study was supported by the Japan Society for the Promotion of Science KAKENHI (ID: 22512878) (to T.M.).

## Ethics Statement

The authors have nothing to report.

## Consent

The authors have nothing to report.

## Conflicts of Interest

Yoshiyuki Akiyama is the Editorial Board member of Lower Urinary Tract Symptoms and the co‐authors of this article. To minimize bias, he was excluded from all editorial decision‐making related to the acceptance of this article for publication. The other authors declare no conflicts of interest.

## Supporting information


**Supporting Information: File** Design drawings of the attachments used to simulate morphological changes of the urethra. All measurements are in millimeters (mm). (A) Normal, (B) One‐side compressed type, (C) both‐side compressed type, (D) Stricture type, (E) Curved type, (F) Strong curved type, (G) Flat type.

**Supporting Information: Movie** Representative findings of high‐speed video recordings obtained using the normal attachment are shown at pump speeds of 200, 300, 400, 500, and 600 RPM.

## Data Availability

Research data are not shared.
