# Supplementary figures and images for: Development of an Imaging‐Based Method for Analyzing Voided Urine Flow Using Conventional and High‐Speed Video Cameras: A Phantom Study
Source: Low Urin Tract Symptoms. 2026 Jul 28;18(5):e70084. doi: 10.1111/luts.70084 (PMC13411646; doi:10.1111/luts.70084)

(A)

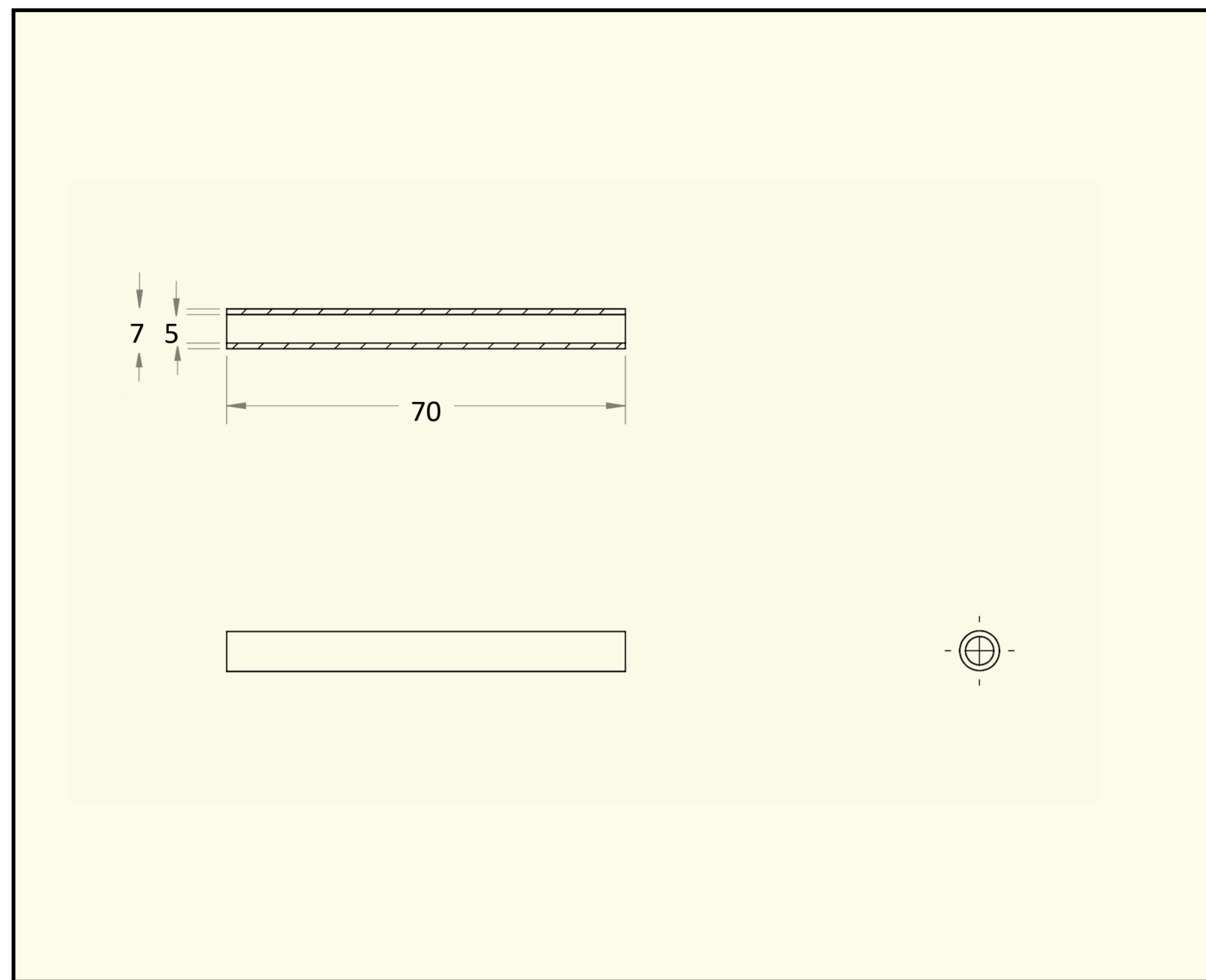

(B)

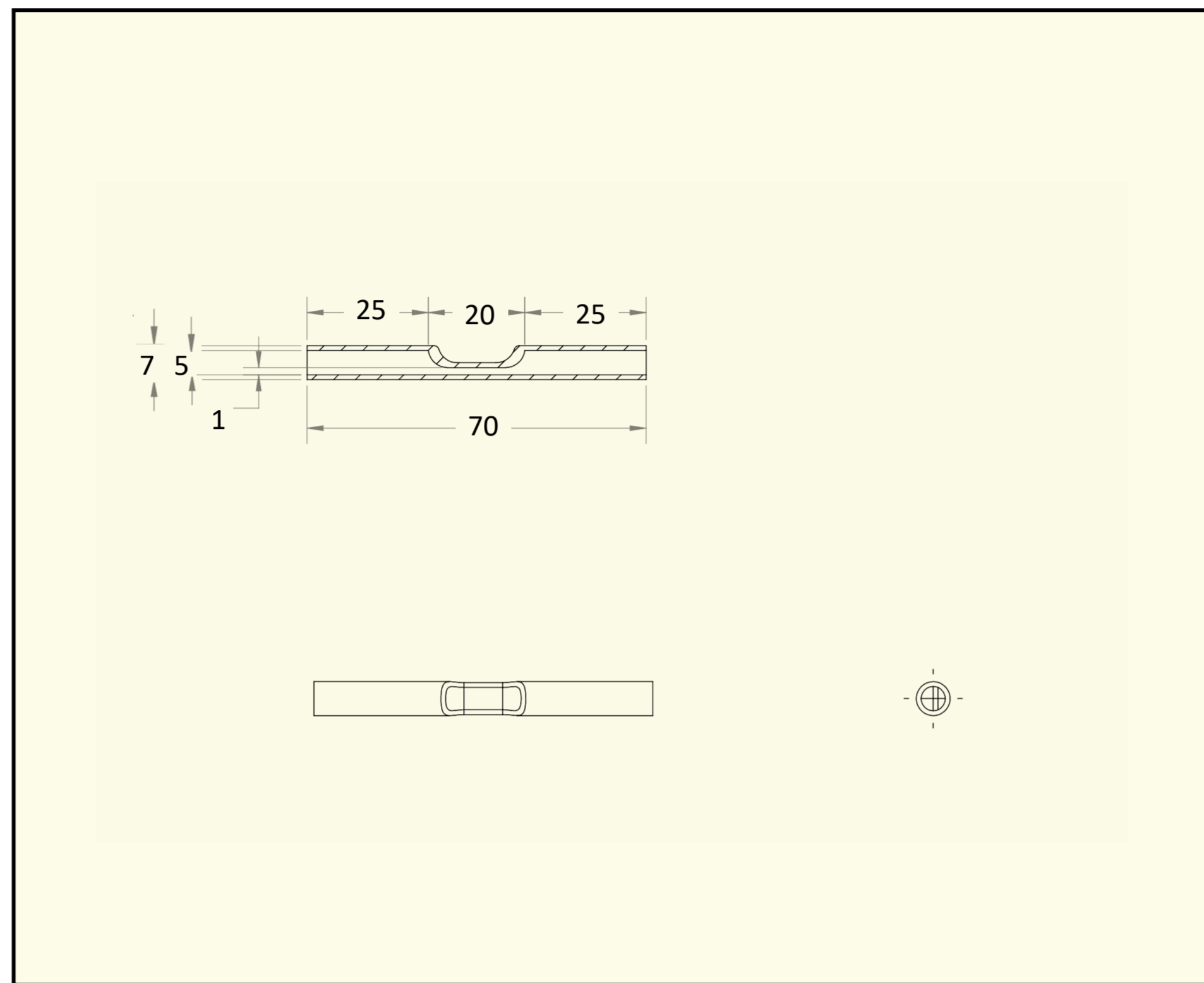

(C)

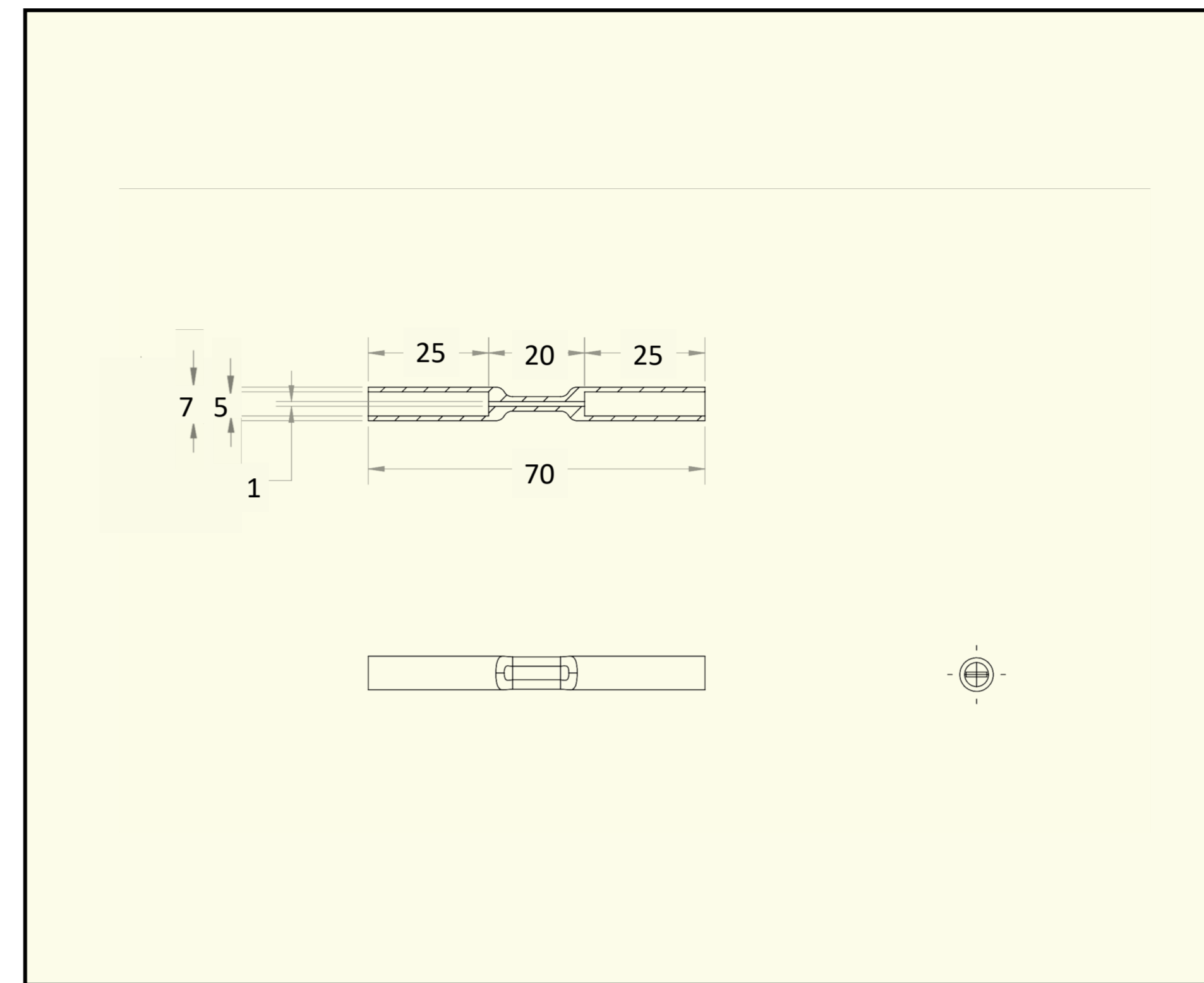

(D)

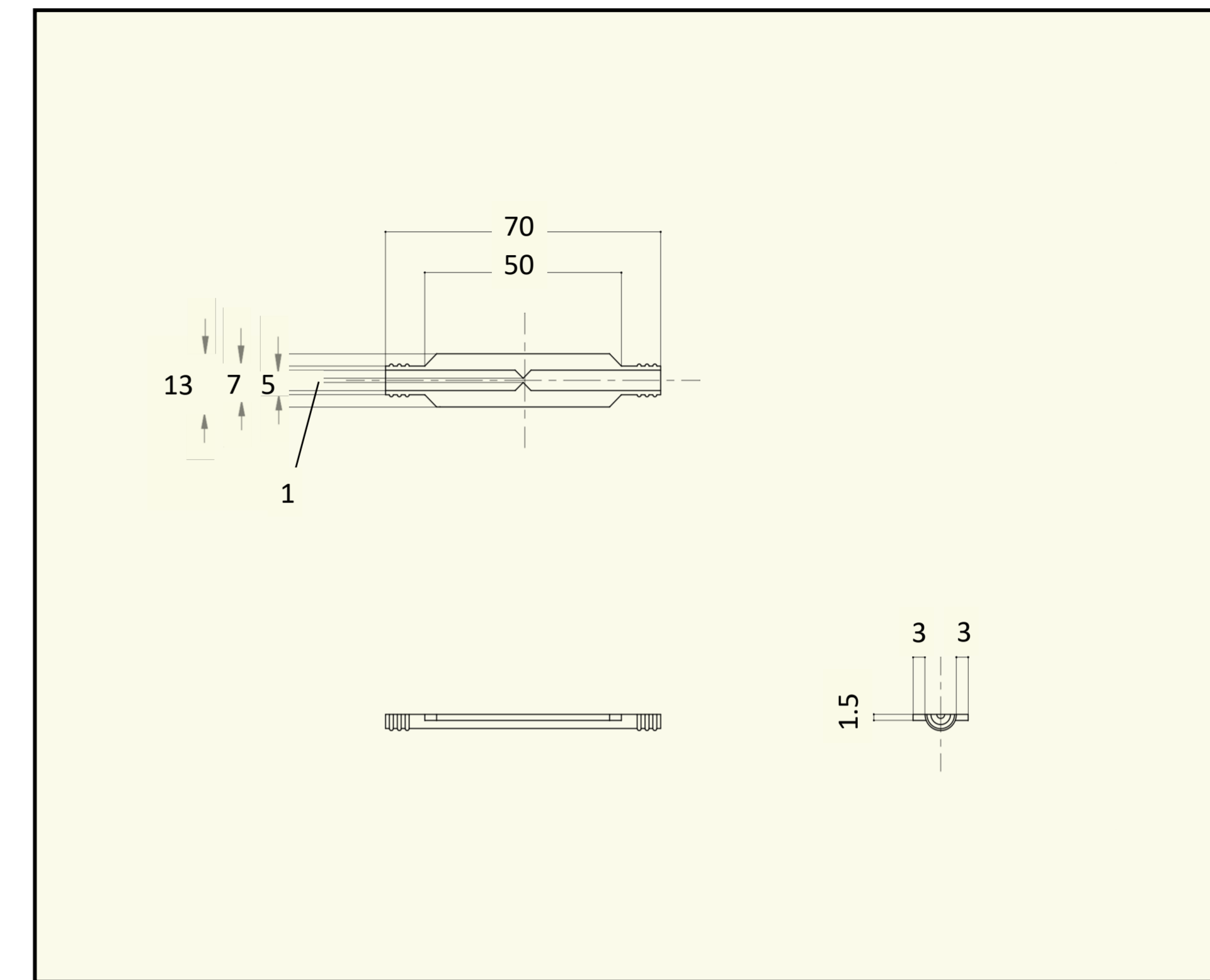

(E)

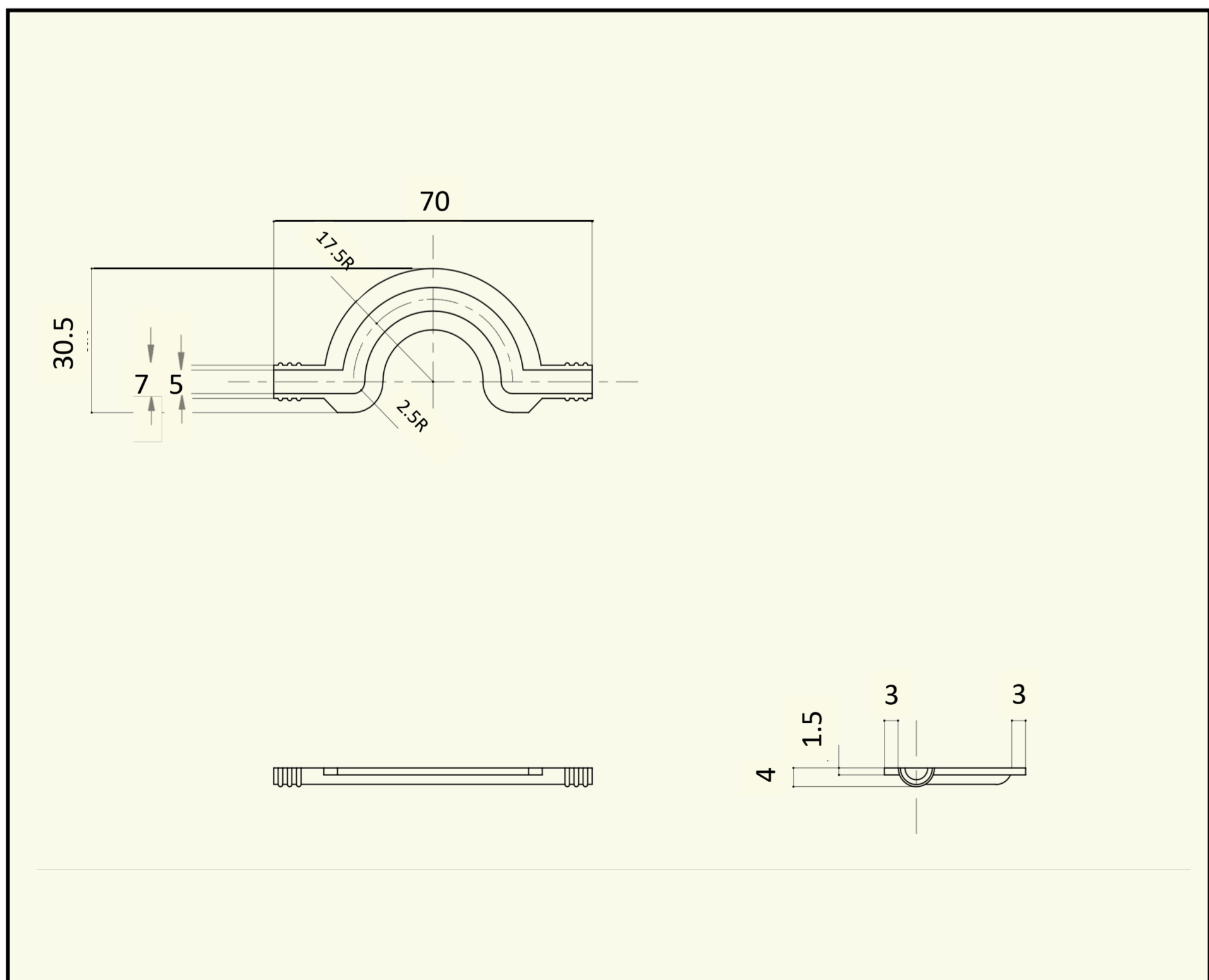

(F)

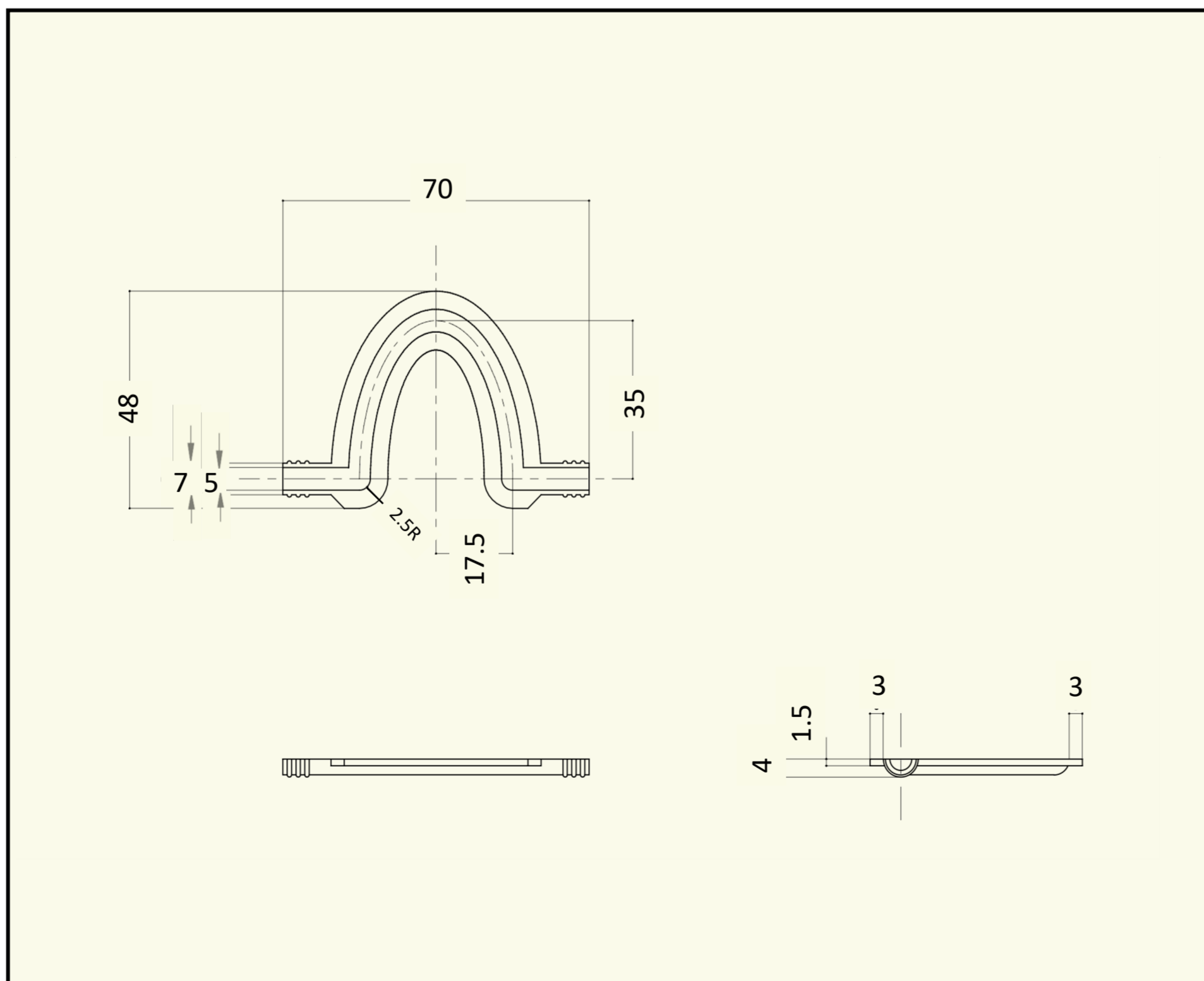

(G)

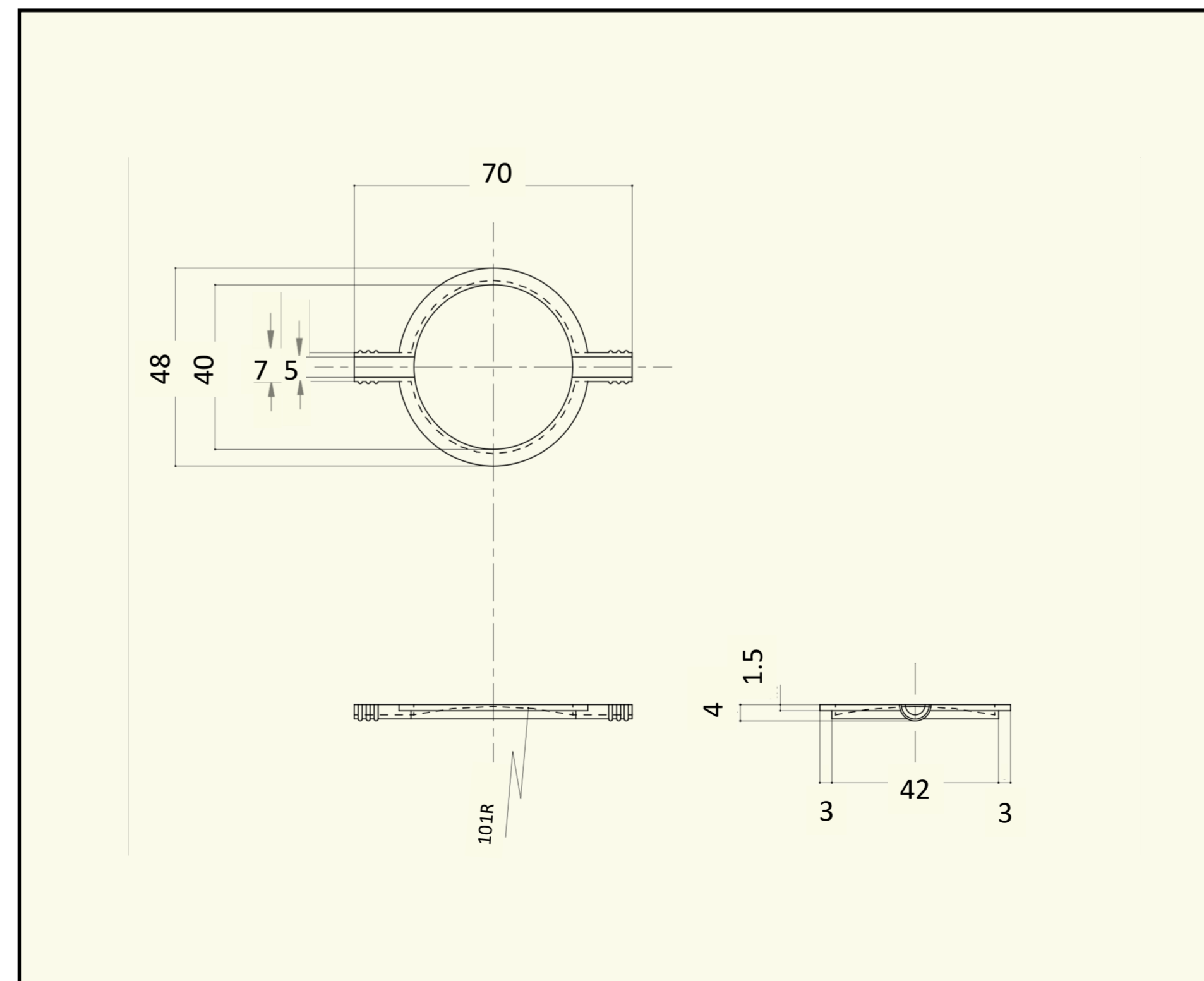

Supplement: Supplementary file 1 — Supporting Information: File Design drawings of the attachments used to simulate morphological changes of the urethra. All measurements are in millimeters (mm). (A) Normal, (B) One‐side compressed type, (C) both‐side compressed type, (D) Stricture type, (E) Curved type, (F) Strong curved type, (G) Flat type. [file LUTS-18-e70084-s001.pdf]
